# One-Step Synthesis of Bifunctional Nickel Phosphide Nanowires as Electrocatalysts for Hydrogen and Oxygen Evolution Reactions

**DOI:** 10.3389/fchem.2021.773018

**Published:** 2021-10-14

**Authors:** Dong Xiang, Biao Zhang, Hongsheng Zhang, Liangping Shen

**Affiliations:** ^1^ School of Mechatronics Engineering, Harbin Institute of Technology, Harbin, China; ^2^ Hubei Yangtze Memory Labs, Hubei University, Wuhan, China; ^3^ Hubei Key Laboratory of Ferro and Piezoelectric Materials and Devices, School of Microelectronics, Hubei University, Wuhan, China

**Keywords:** nickel phosphide, nanowire, hydrogen evolution reaction, oxygen evolution reaction, hydrothermal

## Abstract

The Ni_2_P nanowires were simply synthesized *via* a rapid one-step hydrothermal approach, in which deionized water, red phosphorus, nickel acetate, and hexadecyl trimethyl ammonium bromide were used as the solvent, phosphor and nickel sources, and active agent, respectively. The as-synthesized Ni_2_P nanowire clusters were composed of uniform nanowires with length of about 10 μm and diameter of about 40 nm. The Ni_2_P nanowires exhibited enhanced electrocatalytic activity for both hydrogen evolution reaction and oxygen evolution reaction This work provides good guidance for the rational design of nickel phosphides with unique nanostructures for highly efficient overall water splitting.

## Introduction

Growing energy demands and worsening environmental issues have motivated a large amount of research into developing efficient energy conversion/storage systems for sustainable alternatives (Tan et al., 2020; Ji et al., 2021), e.g., Li-ion batteries (Chen et al., 2016; Zhang et al., 2017a; Li et al., 2017), supercapacitor (Wang et al., 2020; Zhao et al., 2021), water splitting (Wang et al., 2016a; Wang et al., 2016b; Swierk and Mallouk, 2017), and fuel cells (Debe, 2012). Hydrogen generated by water splitting is one of the key strategies for conquering these energy challenges (Kuang et al., 2017). However, the half-reactions of water-splitting, namely hydrogen evolution reaction (HER) and oxygen evolution reaction (OER), suffer from high overpotentials due to sluggish electrode kinetics (Huang et al., 2017). Efficient electrocatalysts, such as noble metal catalysts Pt, Ru, and Ir, are one of the core parts to improve the efficiency of the water decomposition process (Zhou et al., 2016; Zhang et al., 2017b). However, the high cost and scarcity of resources have severely restricted their large-scale applications. Hence, it is fairly urgent to explore efficient, low-cost, and earth-abundant non-noble bifunctional electrocatalysts for HER and OER.

In recent years, nickel-based compounds [oxide (Gong et al., 2014; Qiu et al., 2017; Zhang et al., 2018), hydroxide (Danilovic et al., 2012; Rao et al., 2016), sulfide (Feng et al., 2015; Zhu et al., 2016), and phosphide (Gan et al., 2020; Ji et al., 2021)] have displayed remarkable electrocatalytic activity and stability toward OER and HER, as bifunctional electrocatalysts (Vij et al., 2017). Among them, nickel phosphides could be considered as an efficient and promising candidate in numerous fields of electrochemistry including catalysis (Rao et al., 2016), lithium-ion batteries (Li et al., 2016a), and supercapacitors (Wan et al., 2017). Of note, nickel phosphides (especially metallic-phased phosphide, such as Ni_2_P) are excellent catalysts for HER and OER due to their unique physicochemical properties imparting their high-efficiency and low overpotential (Feng et al., 2014; Liao and Huang, 2017). For example, Matthias Dries et al. (Menezes et al., 2016) reported two remarkably active nickel phosphides that delivered an overpotential of 295 mV for Ni_12_P_5_ and 330 mV for Ni_2_P at 10 mA cm^−2^ for HER, and realized a low potential of 1.64 and 1.58 V at 10 mA cm^−2^ for OER in 1 M KOH, respectively. Ni_x_P_y_ nanocatalysts are highly efficient at driving an overpotential of 1.57 V at 10 mA cm^−2^ in 1.0 M KOH for OER (Li et al., 2016b). Ni_2_P nanoparticles exhibit an overpotential of 0.2 V at 10 mA cm^−2^ in 0.1 M KOH for HER (Li et al., 2015). It is reported that another kind of Ni_2_P nanoparticle delivers an overpotential of 290 mV at 10 mA cm^−2^ in 1 M KOH (Stern et al., 2015). However, the preparation approaches of metal phosphide special nanostructures mainly relies on the high-temperature (over 300°C) oil phase method, e.g., Ni_12_P_5_, Ni_2_P, and Ni_5_P_4_ nanocrystals (320°C) (Pan et al., 2015), and two-step high-temperature (over 300°C) gas–solid reaction, such as CoP nanoneedle (Wang et al., 2016c), CoP film (450°C) (Hellstern et al., 2016), porous Ni_2_P (500°C) (Wang et al., 2016d), FeP nanorods (500°C) (Xiong et al., 2016), and Ni-P porous nanoplates (300°C) (Yu et al., 2016). The low-energy consumption preparations of nickel phosphides with special nanostructures are rarely reported and hard to control, restraining the practical applications of nickel phosphides in electrocatalysis.

The special microstructures of nanowire clusters play a significant role in promoting catalytic activity because of their abundant edge active sites and facilitated charge (including electrons and ions) transfer path (Sivanantham et al., 2016; Tang et al., 2016). In this work, we report a facile one-pot synthesis of Ni_2_P nanowire clusters using the hydrothermal method and the as-prepared Ni_2_P nanowires exhibit enhanced electrocatalytic activity for both HER and OER.

## Experimental Section

### Preparation of Ni_2_P nanowires

In a typical experiment, 2 mmol Ni(CH_3_COO)_2_·4H_2_O, 9 mmol red phosphorus, and 1 mmol hexadecyl trimethyl ammonium Bromide (CTAB) were dissolved in 60 ml pure water. Then, the above solution was transferred into a 100 ml Teflon-lined stainless autoclave, and heated at 195°C for 30 h. After cooling to room temperature, the collected precipitate was filtered and washed with water and ethanol, and then dried overnight.

### Materials Characterization

X-ray diffraction (XRD) patterns of the samples were analyzed by Philips X'Pert PRO (Cu Kα, λ = 0.1542 nm). The microstructures of the samples were examined by scanning electron microscope (SEM, FEI Quanta 200) and the refined microstructures were probed by transmission electron microscopy (TEM, Philips, Tecnai G20). X-ray photoelectron spectroscopy (XPS) spectra were collected on a Kratos AXIS Ultra DLD-600W XPS (a monochromatic Al Kα (1,486.6 eV) as X-ray source).

### Electrochemical Measurement

For the preparation of the working electrode, 5 mg electrocatalyst and 1 mg Ketjen black were dispersed in 968 μL of water/ethanol (volume ratio 4:1) mixture with addition of 32 μL Nafion solution (5 wt%). After ultrasonic dispersion for 30 min, 4 μL of the slurry was drop-cast onto a glassy carbon (GC) electrode with a diameter of 5 mm. The HER and OER tests were carried out by electrochemical workstation (CHI760E, Shanghai Chenhua) and Pine Modulated Speed Rotator with Pt silk as the counter electrode and Ag/AgCl as reference electrode. The polarization curves for HER and OER were obtained at a scan rate of 5 mV s^−1^ under a rotation rate of 1,600 rpm in N_2_-saturated 1 M KOH solution. Electrochemical impedance spectroscopy (EIS) test was performed from a frequency range of 10 kHz to 0.01 Hz at a voltage of −0.4 V (vs. RHE) for HER.

## Results and Discussion

**FIGURE 1:**
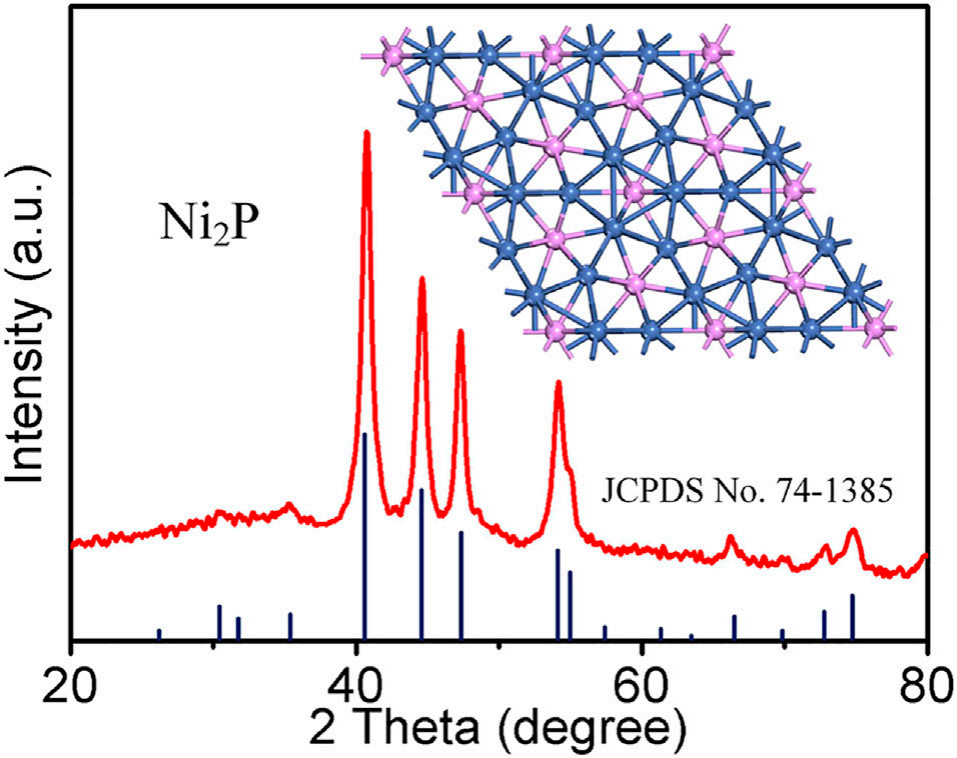
XRD pattern of the as-synthesized Ni_2_P nanowires.

The crystal structure of the as-prepared Ni_2_P was examined by XRD (Figure 1). The diffraction peaks are observed at 30.5, 31.8, 35.3, 40.7, 44.6, 47.4, 54.2, 55.0, 66.4, 72.7, and 74.8°, corresponding to planes (110), (101), (200), (111), (201), (210), (300), (211), (310), (311), and (400). The sample collected at 30 h can be indexed to the hexagonal phase of Ni_2_P (JCPDS 74-1,385) with P-62m space group (the inset in Figure 1 in the atomic structure). There is no superfluous peak, indicating the successful synthesis of pure Ni_2_P.

**FIGURE 2:**
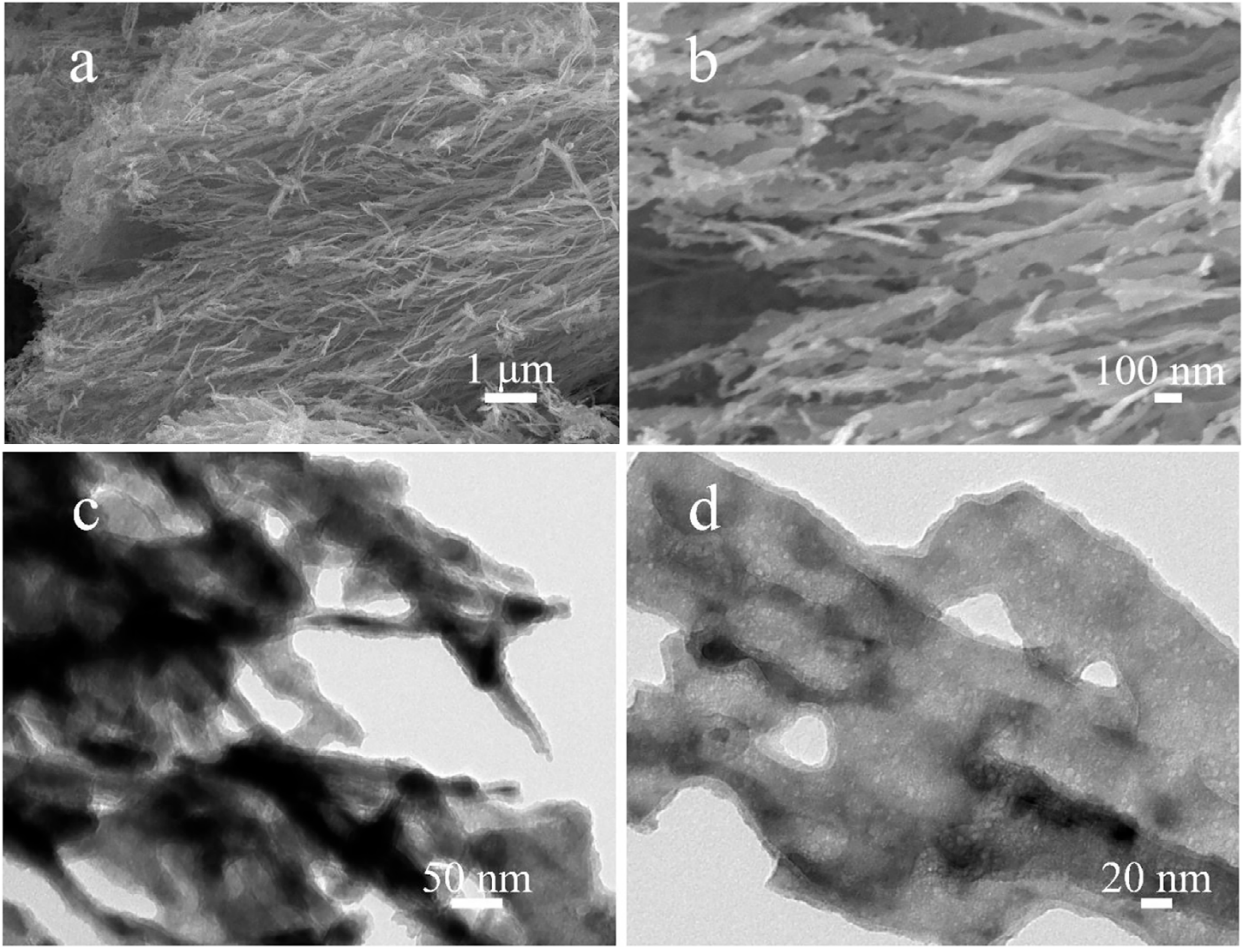
**(A, B)** The SEM images of Ni_2_P nanowires; **(C, D)** The TEM images of Ni_2_P nanowires.

The nanostructures of obtained Ni_2_P nanowires were characterized by SEM and TEM. Figures 2A,B reveal that the Ni_2_P sample is composed of uniform nanowire clusters with lengths of about 10 μm and diameters of about 100 nm. Meanwhile, the orientation of most nanowires is in the same direction as in Figure 2A, and there are numerous hump-like particles on the surface of the nanowires in Figure 2B, exposing a large number of active sites during the electrocatalysis process. A TEM image in Figure 2C shows uniform nanowires of the Ni_2_P sample, and the inside of the nanowires reveals a large number of nanosized holes from the highly magnified TEM image in Figure 2D.

**FIGURE 3:**
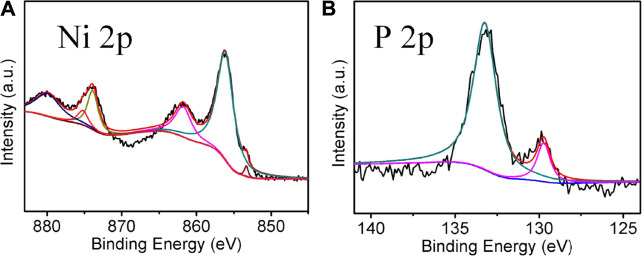
XPS spectra **(A)** Ni 2p, **(B)** P 2p regions for Ni_2_P nanowires.

Figures 3A,B show the core-level XPS spectra of Ni and P elements of Ni_2_P, respectively. As presented, the peaks located at 853.4, 856.3, and 861.9 eV are associated with Ni 2p_3/2_. The peak at 853.6 eV revealed that Ni species in Ni_2_P have a very small positive charge, while the peak at 129.7 eV for P 2p indicates Ni_2_P has a very small negative charge (Wan et al., 2017). In addition, the peaks at 856.3 and 861.9 eV in Ni 2p_3/2_ and the peak at 133.3 eV in P 2p are likely to be ascribed to nickel phosphate formed on the surface of Ni_2_P due to the exposure of the sample to air (Xiao et al., 2016).

**FIGURE 4:**
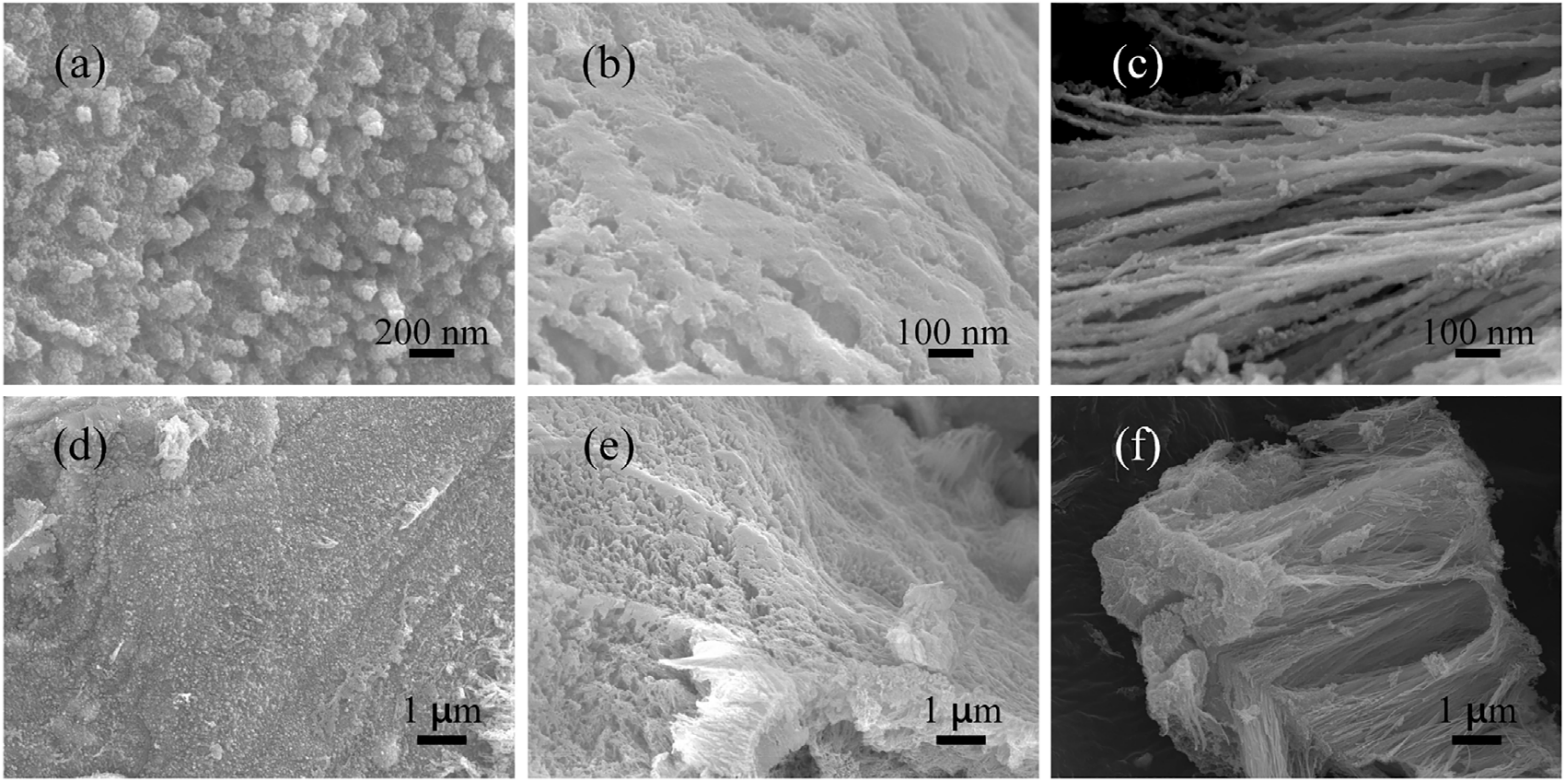
SEM images of obtained samples from different reaction times: **(A, D)** 3 h; **(B, D)** 7 h; **(C, F)** 30 h.

In order to explore the formation mechanism of Ni_2_P nanowires, a series of samples that underwent different reaction times were collected. The SEM images of the sample collected at 3 h in Figures 4A,D show the surface of a block has a uniform arrangement of projections with a length of about 200 nm and a diameter of about 40 nm. The sample obtained at 7 h shows a larger cavity than that at 3 h as shown in Figures 4B,E. Figures 4C,F reveal that the sample obtained at 30 h is composed of nanowire clusters with the same orientation and length of about 10 μm and a diameter of about 100 nm. Taking red phosphorus as the phosphorus source and nickel acetate as the nickel source during hydrothermal reaction, the Ni_2_P nanowires were successfully synthesized. At first, red phosphorus is difficult to dissolve in deionized water. With the hydrothermal process (process 1), red phosphorus was gradually decomposed to generate phosphine, and then the phosphine reacted with nickel ions in solution and nucleation occurs at the surface of the block. Following this (process 2), the block of red phosphorus was gradually consumed, and the nanowires gradually increase. Finally (process 3), the Ni_2_P nanowires were formed, accompanied with red phosphorus and nickel ions depleting.

**FIGURE 5:**
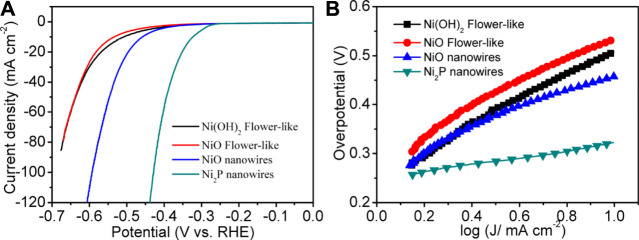
**(A)** LSV curves of Ni_2_P nanowires, NiO nanowires, Ni(OH)_2_ flower-like at 5 mV s^−1^ in 1 M KOH from −0.7–0 V vs. RHE. **(B)** Tafel plots of the HER activity.

Figure 5A shows the linear sweep voltammogram (LSV) curve of Ni_2_P nanowire catalysts at 5 mV s^−1^ after 20 cycles of cyclic voltammogram (50 mV s^−1^) activation. For comparative analysis, the LSV curves of the samples, *i.e.*, Ni_2_P nanowires, Ni(OH)_2_ flower-like nanostructures, and NiO flower-like nanostructures (SEM images as shown in Supplementary Figure S1), were also measured at 5 mV s^−1^ with the same mass loadings of 0.175 mg cm^−2^. The polarization curves of Ni_2_P nanowires exhibit a remarkable electrocatalytic activity for HER with a small onset potential and overpotential (η) to reach a current density of 10 mA cm^−2^. The ranking of the overpotentials for those catalysts is: Ni_2_P nanowires (320 mV) < Ni_2_P nanowires (458 mV) < Ni(OH)_2_ nanoflowers (512 mV) < NiO nanoflowers (535 mV). It is clear that Ni_2_P nanowires exhibit the highest electrocatalytic activity toward HER. The Tafel slope for the Ni_2_P nanowires catalyst was about 73 mV dec^−1^ (Figure 5B), much smaller than those of the NiO nanowires (157 mV dec^−1^), flower-like Ni(OH)_2_ (234 mV dec^−1^), and flower-like NiO (213 mV dec^−1^), which further confirmed the superior electrocatalytic HER kinetics of Ni_2_P nanowires.

**FIGURE 6:**
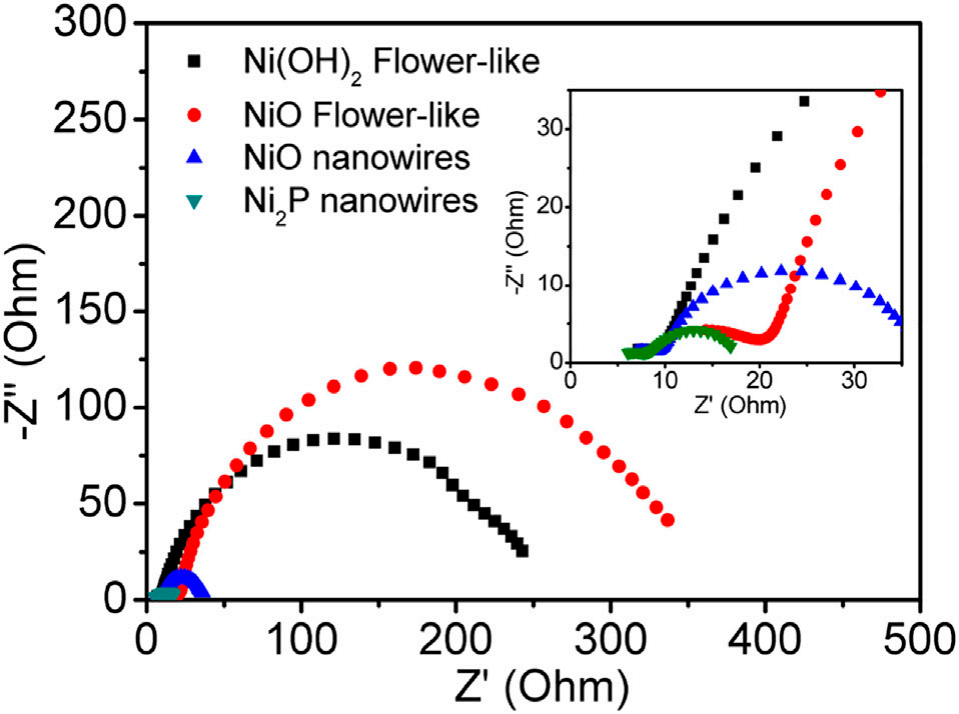
Electrochemical impedance spectrum of Ni_2_P nanowires.

To further understand the reason for the excellent electrocatalytic HER activity of Ni_2_P nanowires, EIS analysis was carried out (Figure 6). The charge transfer resistance under high frequency of Ni_2_P nanowire is low, which further implies its higher conductivity. The lower charge transfer resistance and higher diffusion of electrolyte ions indicate good electronic conductivity and high OH^−^ ion transfer speed in the interface of active materials/electrolyte. The aforesaid electrochemical performances reveal that Ni_2_P nanowire clusters are an efficient and sturdy electrocatalyst for HER in strongly basic media.

**FIGURE 7:**
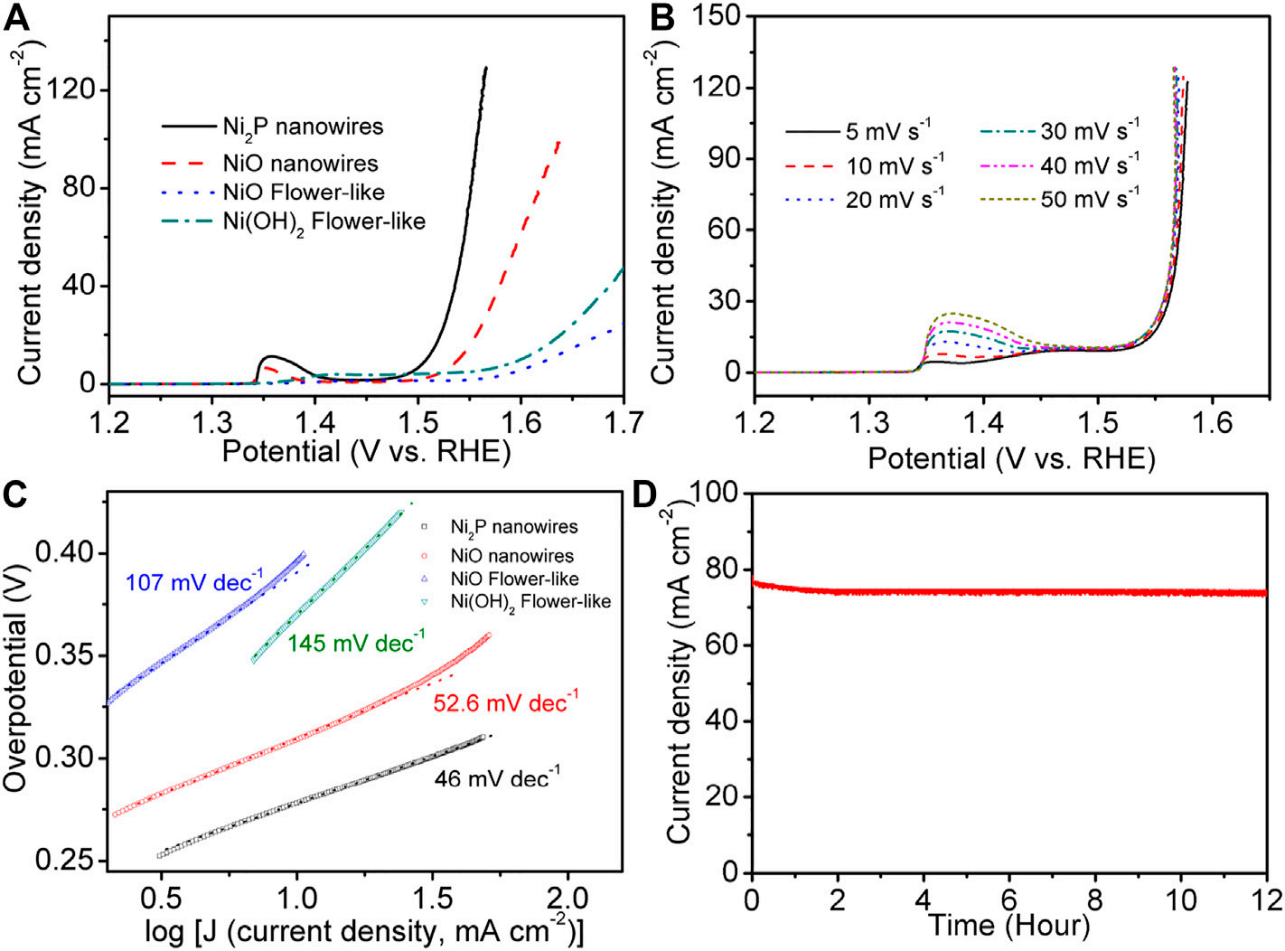
**(A)** LSV curves of Ni_2_P nanowires, NiO nanowires, Ni(OH)_2_ flower-like at 5 mV s^−1^ in 1 M KOH. **(B)** LSV curves of Ni_2_P nanowires at different scan rates. **(C)** Tafel plots of the OER activity. **(D)** The stability of the Ni_2_P nanowires tested in amperometric i-t curve at 1.7 V vs. RHE.

Figure 7A shows the linear sweep voltammograms (LSV) curve of Ni_2_P nanowire catalyst at 5 mV s^−1^ after 20 cycles of cyclic voltammogram (at the scan rate of 50 mV s^−1^) activation. For comparative analysis, the LSV curves of NiO nanowires, Ni(OH)_2_ flower-like, and NiO flower-like catalysts were also measured at 5 mV s^−1^ with the same mass loadings of 0.175 mg cm^−2^. The polarization curves of Ni_2_P nanowires exhibit a higher current density and more negative OER overpotential of 280 mV than those of NiO nanowires (310 mV), Ni(OH)_2_ flower-like (370 mV), and NiO flower-like (390 mV). In order to further study the polarization property, the LSV curves of Ni_2_P nanowires at different scan rates were displayed in Figure 7B. It indicates that the polarization curves have no difference in addition to the intensity of the oxidation peaks. This oxidation peak is also reversible for Ni_2_P nanowires as observed from the cyclic voltammogram (Supplementary Figure S2). The Tafel slope for the Ni_2_P nanowires catalyst was about 46 mV dec^−1^ (Figure 7C), much smaller than those of the NiO nanowires (52.6 mV dec^−1^), flower-like Ni(OH)_2_ (145 mV dec^−1^), and flower-like NiO (107 mV dec^−1^), which further confirmed the superior electrocatalytic OER kinetics of Ni_2_P nanowires. The stability of the Ni_2_P nanowires for OER was tested in amperometric i-t curve at 1.7 V (vs. RHE) for 12 h (Figure 7D), indicating its good durability.

## Conclusion

In summary, we firstly synthesized Ni_2_P nanowires using a facile one-step hydrothermal approach. The as-synthesized Ni_2_P is composed of nanowire clusters with a uniform length of about 10 μm and a diameter of about 40 nm. There are a large number of nanoparticles on the surface of the nanowires, providing a large number of active sites during the electrocatalysis process. The overpotential of Ni_2_P nanowires is 320 mV and clearly demonstrates the Tafel slope of 73 mV dec^−1^ for HER. Meanwhile, the Ni_2_P nanowires show excellent electrocatalytic OER activity with overpotential of 1.51 V (vs. RHE) and Tafel slope of 46 mV dec^−1^. This work provides good guidance for the rational design of nickel phosphides with unique nanostructures for highly efficient overall water splitting.

## Data Availability

The raw data supporting the conclusion of this article will be made available by the authors, without undue reservation.
