# Supplementary material for: One-Step Synthesis of Bifunctional Nickel Phosphide Nanowires as Electrocatalysts for Hydrogen and Oxygen Evolution Reactions
Source: Front Chem. 2021 Oct 14;9:773018. doi: 10.3389/fchem.2021.773018 (PMC8552028; doi:10.3389/fchem.2021.773018)
Supplement: Supplementary file 1 [file DataSheet1.pdf]

Supplementary Materials

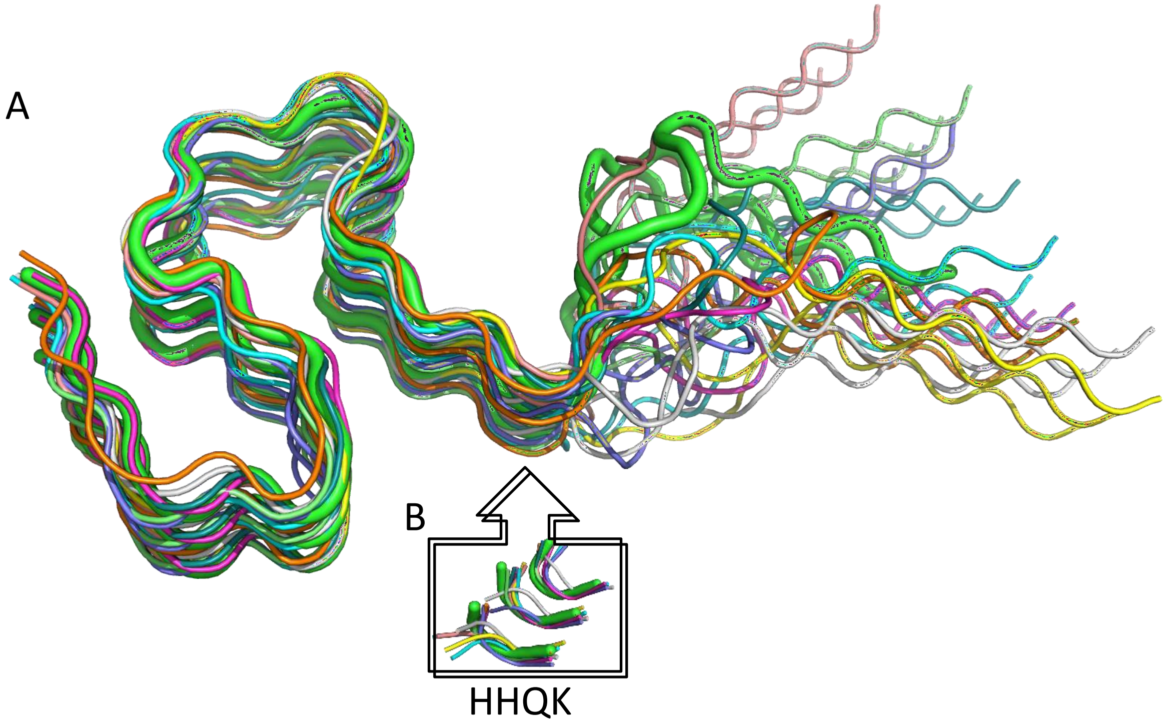

Supplementary Figure 1: Structural differences of Aβ peptide (A) and HHQK site (B) defined in solution NMR models. The lowest potential energy model is represented as a tube cartoon diagram. The other models are depicted using loop cartoon representations.

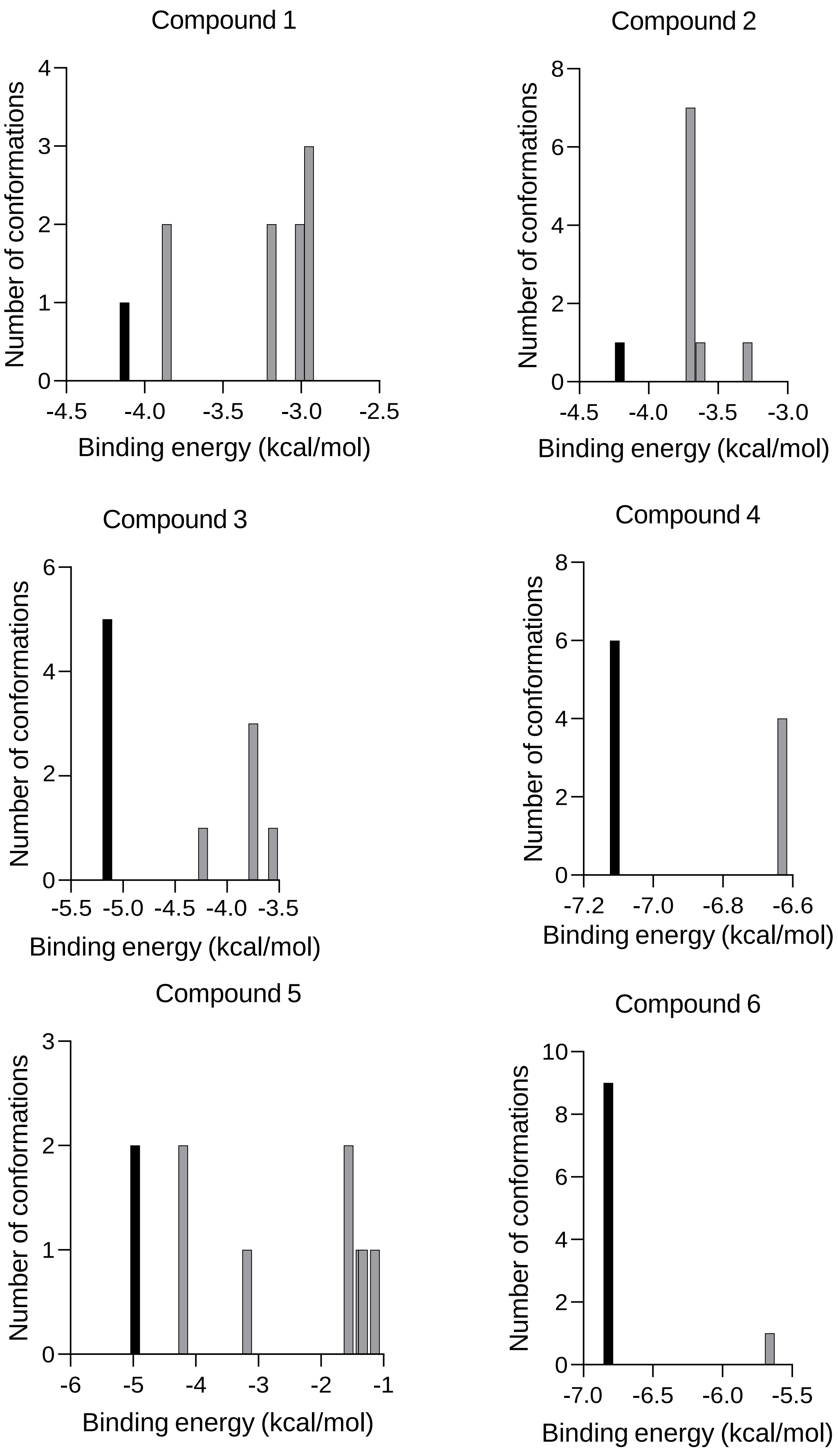
Compound 1
Number of conformations
Binding energy (kcal/mol)
Compound 2
Number of conformations
Binding energy (kcal/mol)
Compound 3
Number of conformations
Binding energy (kcal/mol)
Compound 4
Number of conformations
Binding energy (kcal/mol)
Compound 5
Number of conformations
Binding energy (kcal/mol)
Compound 6
Number of conformations
Binding energy (kcal/mol)

Compound 7

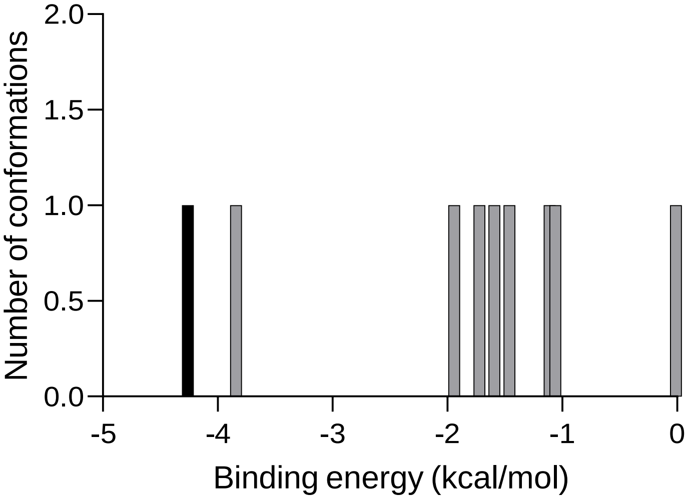

Compound 8

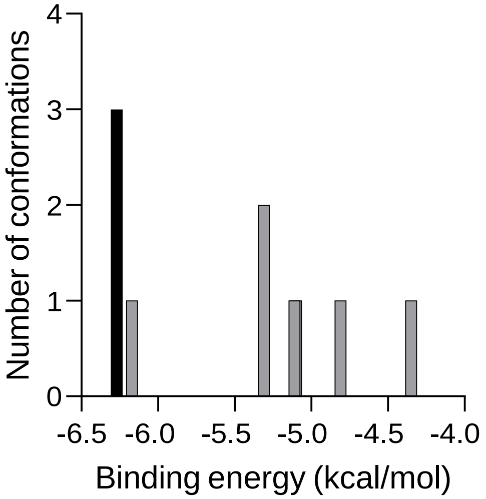

Compound 9

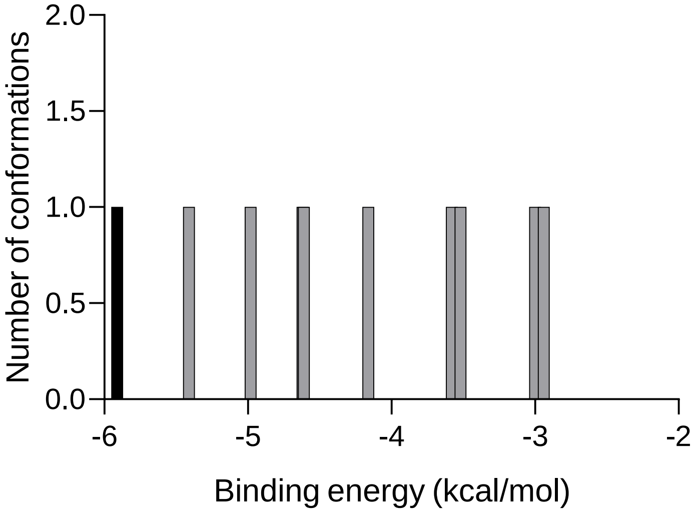

Compound 10

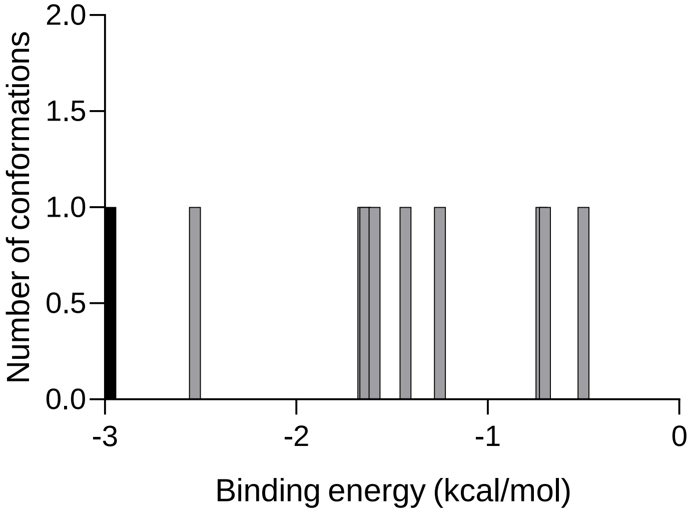

Compound 11

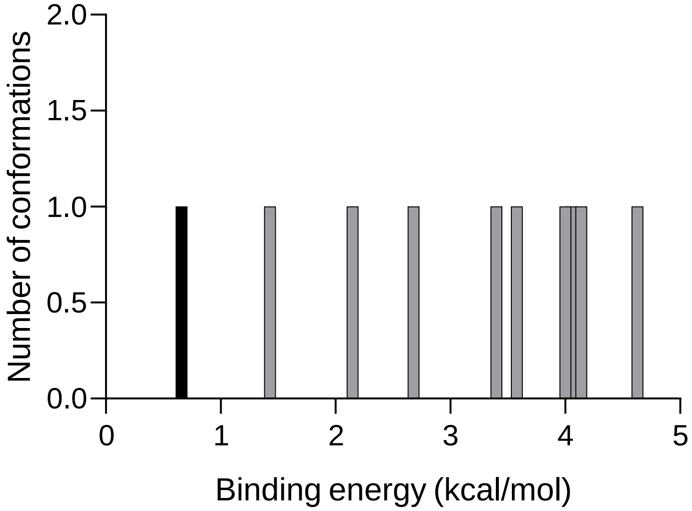

Compound 12

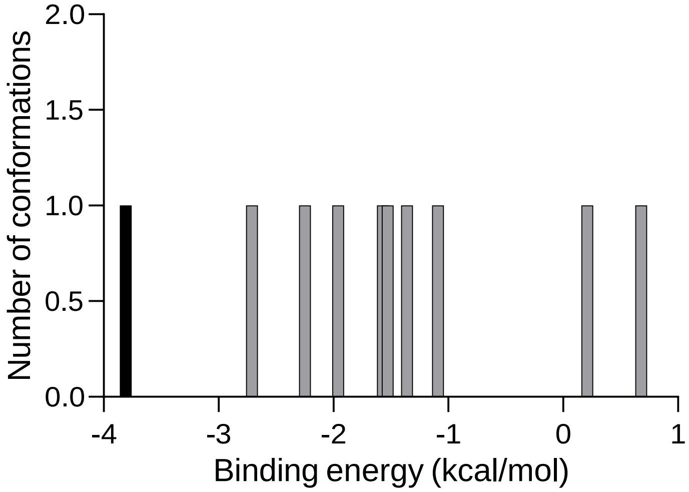

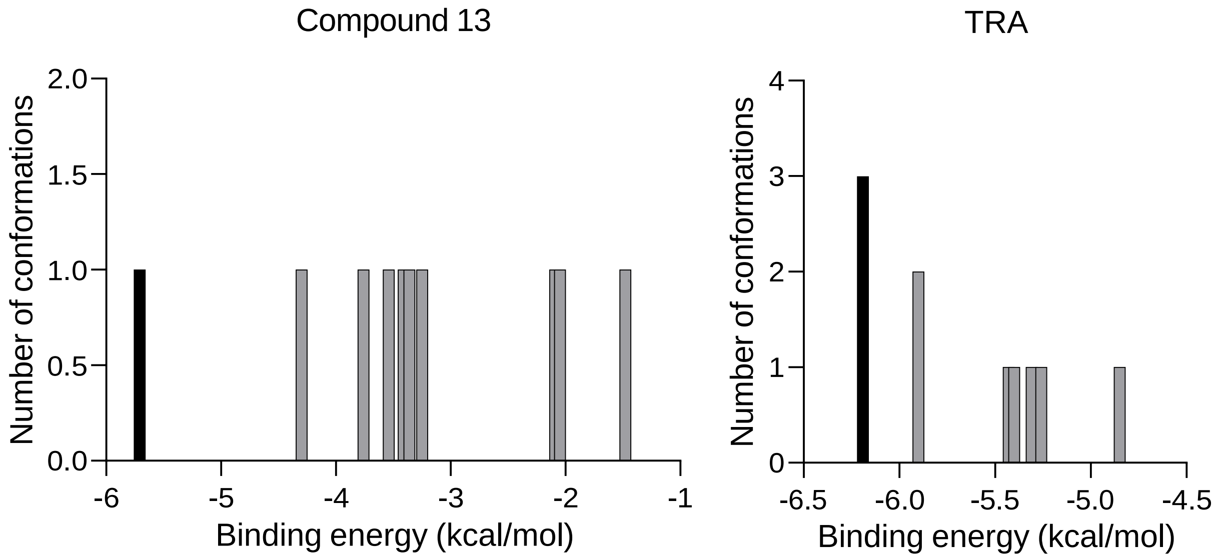

Supplementary Figure 2: Conformational clustering of molecular docking results for analyzed compounds. Cluster allocation was processed with an RMS tolerance of 2.0 Å between conformations, respectively. The lowest-energy cluster is marked in black.

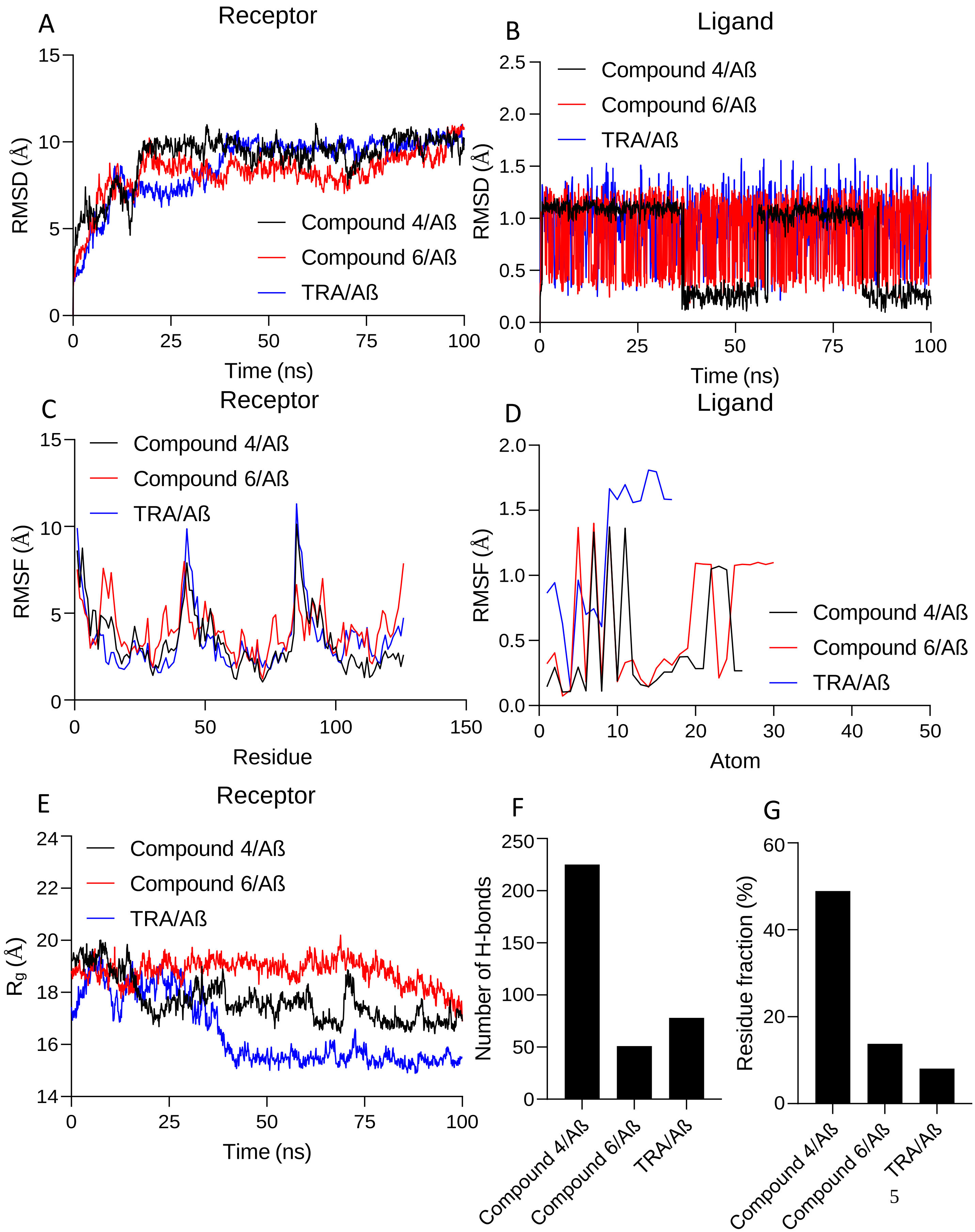
A
Receptor
RMSD (Å)
Time (ns)
Compound 4/Aß
Compound 6/Aß
TRA/Aß
B
Ligand
RMSD (Å)
Time (ns)
Compound 4/Aß
Compound 6/Aß
TRA/Aß
C
Receptor
RMSF (Å)
Residue
Compound 4/Aß
Compound 6/Aß
TRA/Aß
D
Ligand
RMSF (Å)
Atom
Compound 4/Aß
Compound 6/Aß
TRA/Aß
E
Receptor
Rg (Å)
Time (ns)
Compound 4/Aß
Compound 6/Aß
TRA/Aß
F
Number of H-bonds
Compound 4/Aß
Compound 6/Aß
TRA/Aß
G
Residue fraction (%)
Compound 4/Aß
Compound 6/Aß
TRA/Aß

Supplementary Figure 3: RMSD (A, B), RMSF (C, D), Rg (E), number of H-bonds (F) and the fraction of residues involved in H-bonding (G) calculated using receptor-ligand complexes of analyzed compounds and Aβ peptide during 100 ns MD simulation.
